# Supplementary material for: Rapid turnover of life-cycle-related genes in the brown algae
Source: Genome Biol. 2019 Feb 14;20:35. doi: 10.1186/s13059-019-1630-6 (PMC6374913; doi:10.1186/s13059-019-1630-6)
Supplement: Supplementary file 3 — Protein alignments, corresponding Gblocks html file and CDS sequences for the lineage conserved gametophyte-biased genes. (ZIP 2109 kb) [file 13059_2019_1630_MOESM3_ESM.zip › Con_GA_alignments/Ec-06_008340.aa_ali.fasta-gb.htm]

Ec-06\_008340.aa.fasta.trans.aa\_ali.fasta


## Gblocks 0.91b Results

Processed file: **Ec-06\_008340.aa.fasta.trans.aa\_ali.fasta**  
Number of sequences: **2**  
Alignment assumed to be: **Protein**  
New number of positions: **348** (selected positions are underlined in blue)

```
                         10        20        30        40        50        60
                 =========+=========+=========+=========+=========+=========+
Ec-06_008340     MDLTKAVLKKICRDSGLYSSPSLNDKLYLHYKGIRQIQNLEEYTGLRVLWLEGNGLSKLE
MSTRG.26060.1    MDLTKAMLRTMCRDNGQYSSPALNDKLYLHYKGIRKIQNLDEYTGLRVLWLEGNGLCKLE
                 ############################################################


                         70        80        90       100       110       120
                 =========+=========+=========+=========+=========+=========+
Ec-06_008340     GMEAQTQMKTLYAHENLIEKIEGLDSF---------------------------------
MSTRG.26060.1    GMRAQTQLKTLYAHENLIEKIEGLESFRELDTLNLSKNFVRRVENIGHLGRLTSLLLANN
                 ###########################                                 


                        130       140       150       160       170       180
                 =========+=========+=========+=========+=========+=========+
Ec-06_008340     ----------------LETLDVQHNRINDPDVVDIVSAMHDLRVLYLQGNPVVKSIRHYR
MSTRG.26060.1    HLESAEDVRQVLQCPSIQTLDLQHNRLNDPHVVDVVSAMQDLRVLYLRGNPVVKLVRHYR
                                   ##########################################


                        190       200       210       220       230       240
                 =========+=========+=========+=========+=========+=========+
Ec-06_008340     KTLVSKCATLKYLDDRPVFDDERCRCAAWSKGMAEGGISRAQEAEREEMAKIRESKRAAE
MSTRG.26060.1    KTLVSKCVSLKYLDDRPVFDEERRRCAAWSKGMTDGGISRAQEAEREEMARIRASRQAAE
                 ############################################################


                        250       260       270       280       290       300
                 =========+=========+=========+=========+=========+=========+
Ec-06_008340     QRQYLAFEQMMREGLEAKKQAKPQHESLSENAEESEASGQPEPPVVNDSSQVSISTVQEG
MSTRG.26060.1    QRQYLAFEQMMREGLTAKQKGEKQFE--GE--------NPTSPSIVNHGGQASTARLAIG
                 ##########################                ##################


                        310       320       330       340       350       360
                 =========+=========+=========+=========+=========+=========+
Ec-06_008340     QESPLCDTSNEHCEVGNTGSSIGGDGTSGQQETLPLPPLPPPQNVVLGAIVQMNPRGQQK
MSTRG.26060.1    KEAMLPD---------DVSRSASGDGKPRRDLTPLLPPPSP------GKMTLSAESSE--
                 #######             #####################                   


                        370       380       390       400       410       420
                 =========+=========+=========+=========+=========+=========+
Ec-06_008340     SMVSNTMEERNTRVPRLVNEFSGEDIIPFTDCETVRNARQSRAARILAPSTST-HRAPSS
MSTRG.26060.1    -------QTLACSVP-PINNFSGEDILTVSDCETVKQARQRRVARILATSVAVRNVQPAN
                                   ################################          


                        430       440       450       460       470       480
                 =========+=========+=========+=========+=========+=========+
Ec-06_008340     DATDSPEGSGIRRSPGLDHVEPLDDDVFDLTKLSNDVDEEIRAFEAESQRLREERILQNT
MSTRG.26060.1    TKRPTTQQRVVVDSP--VKAGPLNENDLDLKQLSACVGEEIRSFEEKSALLREERMAQNA
                                      #######################################


                        490       500       510       520
                 =========+=========+=========+=========+========
Ec-06_008340     P-PPPPATVKKQEVVEVGCDGNVEVTYQEHVAEFCQEQA--TNFEELD
MSTRG.26060.1    PLPPPAAAVRPSTISGIMWNKGMDVTVGMDVLGDRHEPAAVTNFETLD
                 #                       ###############
```

```
Parameters used
Minimum Number Of Sequences For A Conserved Position: 2
Minimum Number Of Sequences For A Flanking Position: 2
Maximum Number Of Contiguous Nonconserved Positions: 8
Minimum Length Of A Block: 10
Allowed Gap Positions: None
Use Similarity Matrices: Yes
```

```
Flank positions of the 7 selected block(s)
Flanks: [1  87]  [139  266]  [283  307]  [321  341]  [379  410]  [442  481]  [505  519]  

New number of positions in Ec-06_008340.aa.fasta.trans.aa_ali.fasta-gb:  348  (65% of the original 528 positions)
```
